# Risk factors associated with occurrence of placental malaria in a population of parturients in Abeokuta, Ogun State, Nigeria

**DOI:** 10.5281/zenodo.10870262

**Published:** 2015-06-22

**Authors:** Ayodele S. Babalola, Oluwafunmilayo A. Idowu, Sammy O. Sam-Wobo, Eniola Fabusoro

**Affiliations:** 1 Department of Biological Sciences, Federal University of Agriculture, Abeokuta, Nigeria; 2 Department of Agricultural Extension and Rural development, Federal University of Agriculture, Abeokuta, Nigeria

## Abstract

**Background:**

Placental malaria has long been acknowledged as a complication of malaria in pregnancy, and has been associated with poor pregnancy outcome in malaria-endemic areas. This study was conducted to determine the risk factors associated with occurrence of placental malaria in a population of parturients in Abeokuta Ogun State, Nigeria.

**Materials and Methods:**

Maternal and placenta blood samples were collected from 211 parturients. Blood films were prepared, stained with 10% Giemsa and microscopically analysed for the presence of parasites. Demographic characteristics were recorded in case record forms. Chi-square tests and a regression model were computed to analyse risks, using SPSS version 16.0.

**Results:**

Overall, 40.8% (86 of 211) of the parturients had malaria at the time of delivery, with 19.0% (40 of 211) having placental malaria. We identified being within the age range of 18-22 years [OR = 4.4, 95% CL = 1.1-17.4, *P* = 0.046], being primigravid [OR = 2.1, 95% CL = 0.9-5.1, *P* = 0.028] and living in a congested apartment [OR = 1.6, 95% CL = 0.4-6.0, *P* = 0.029] as significant risk factors for placental malaria. Non-usage of intermittent preventive treatment (IPT) [OR = 2.6, 95% CL = 1.2-5.4, *P* = 0.018], long-lasting insecticidal nets (LLINs) [OR = 2.7, 95% CL = 1.3-5.5, *P* = 0.005] were also risk factors for placental malaria.

**Conclusions:**

In Abeokuta, the proper use of LLIN and IPT for pregnant women is essential to curb the scourge of malaria, associated risks and poor pregnancy outcomes.

## 1 Introduction

Malaria continues to remain one of the most severe and complex health challenges facing the vast majority of tropical and sub-tropical countries around the world. The population groups most at risk of malaria include pregnant women and children under the age of five [1]. During pregnancy, the acquired anti-malarial immunity of a woman residing in a malaria-endemic area is affected due to up regulation of the VAR2CSA gene in local *Plasmodium falciparum* [2]. The risk of malaria increases threefold during the second and third trimesters, and fourfold during the initial two post-partum months [3]. This increased risk of malaria is related to an alteration in the balance of Th1-and Th2-related immune factors [4].

In clinical practice, maternal peripheral parasitaemia is used to detect malaria during pregnancy. However, whereas peripheral parasitaemia may remain below the levels of microscopic detection, these parasites are detectable in the placenta [5,6]. Although the mechanism for placental parasitisation is not yet fully known, certain parasite strains have been increasingly detected in pregnant women. It has been suggested that these strains are able to selectively infect the placenta due to their ability to adhere to the chondroitin sulphate A (CS-A) receptor on the syncytiotrophoblast [5,6].

Placental malaria has long been recognised as a complication of malaria in pregnancy in areas of stable transmission, and is particularly frequent and more severe in primigravidae [7]. Placental malaria is a serious issue in sub-Saharan Africa and is associated with poor pregnancy outcomes, such as low birth weight (LBW), stillbirth and preterm delivery.

Studies have shown that *Plasmodium* parasites can cross the placenta, leading to mother-to-child transmission of malaria in a condition known as congenital malaria [8,9]. Moreover, during delivery, blood vessels may rupture, leading to a mixing of maternal and foetal blood, and transmission of parasites from mothers to children [10]. Studies in Africa have shown that at least 7-10% of newborns have malaria parasite-infected placentas, and a significant part of the transmission of parasites from the mother to the child occurs well before the time of delivery [11,12]. It is also estimated that 6% of all infant deaths in malaria-endemic areas are a result of malaria infection that took place during the child’s prenatal life [13]. As pitiful as this situation is, the exact mechanisms for placenta parasitisation and transplacental transmission of malaria parasites are not well understood [5]. Therefore, it is very important to identify the risk factors for placental malaria so that pregnant women can make lifestyle choices to reduce the chance of developing malaria. Therefore, this study was conducted to determine risk factors associated with the occurrence of placental malaria in a population of parturients in Abeokuta Ogun State, Nigeria.

## Materials and Methods

### 2.1 Study area

The research was carried out in Abeokuta, the capital of Ogun State in Nigeria. Abeokuta is located within longitude 7°15’ North and latitude 3°21’ East. Two hospitals, Oba-Ademola Maternity Hospital (coordinates 7° 10’20.8”N, 3°2’29.6”E) and State Hospital Sokenu Ijaiye (coordinates 7°09’11.9”N, 3°21’10.9”E), were involved in this study. The two hospitals are among the most frequently patronised health facilities in Abeokuta.

### 2.2 Criteria for selection and study population

Only pregnant women in labour (parturients) were included in this study. A total of 211 parturients were randomly selected from the two hospitals, using a formula according to Yamane [14]. The sample population was selected irrespective of age, educational background, marital status, occupation, and cultural or religious affiliations.

### 2.3 Ethical clearance/informed consent

The Research and Ethics Committee of the State Hospital Sokenu Ijaiye provided ethical clearance for the study. All medical personnel of the selected hospitals and the participants were fully briefed on the objectives of the study. Only participants who gave their consent were enrolled in the study.

### 2.4 Collection of placenta blood samples and questionnaire administration

Maternal blood and placenta blood were collected as described by [15]. Relevant maternal demographic and clinical characteristics were recorded with the aid of a questionnaire. Information obtained included: age, gravidity, education, and use of intermittent preventive treatment (IPT) and/or long-lasting insecticidal nets (LLINs).

### 2.5 Laboratory procedures

Thick and thin smears were prepared and stained with 10% Giemsa as described by Cheesbrough [16]. The blood films were examined microscopically using 100× objectives with oil immersion. Malaria diagnosis was based on identification of asexual stages of *Plasmodium spp.* on the thick blood film, while thin smears were used for species identification. Slides were declared negative after observing at least 100 high-power fields without detecting any parasites. Parasite density was calculated by estimating parasite numbers/μl of blood from the thick film as described by Greenwood & Armstrong [17]. Parasite densities were grouped as low (≤500 parasites/μl), moderate (501-5000 parasites/μl) or high (>5000 parasites/μl).

### 2.6 Statistical analysis

Chi-square tests and logistic regression were used to analyse data, and a probability value of *P*<0.05 was regarded as significant, using SPSS version 16.0.

## 3 Results

### 3.1 Study population

The mean age of the parturients was 28.5±0.3 years. The majority were within the age range of 28-32 years [94 (44.5%)], followed by the age group 23-27 years. The highest proportion of the parturients were multigravids [87 (41.2%)], followed by primigravids [67 (31.8%)] and secundigravids [57 (27.0%)], respectively. The majority of the parturients had tertiary education and were mainly traders by occupation, followed by teachers and civil servants (Table 1).

**Table 1.** Characteristics of the respondents

Variable	Frequency (*N*)	Percentage (%)
**Age (years)**		
18 to 22	22	10.4
23 to 27	53	25.1
28 to 32	94	44.5
33 and above	42	19.9
Total	**211**	**100.0**
**Gravidity**		
Primigravid	67	31.8
Secundigravid	57	27.0
Multigravid	87	41.2
Total	**211**	**100.0**
**Education level**		
Primary	39	18.5
Secondary	77	36.5
Tertiary	95	45.0
Total	**211**	**100.0**
**Occupation**		
Professionals	5	2.3
Civil servant	33	15.6
Artisan	29	13.8
Nurse	6	2.8
Student	21	10.0
Teacher	38	18.0
Trader	79	37.4
Total	**211**	**100.0**

### 3.2 Malaria prevalence

Overall, 40.8% (86 of 211) of the parturients had malaria at the time of delivery, with 19.0% (40 of 211) having malaria parasites in their placental blood (placental malaria), with mean maternal peripheral and placenta blood malaria parasite densities of 2597±274 and 3922±561 parasite/μl of blood, respectively. The prevalence of placental malaria decreased with age and gravidity (Table 2). More than 30% of the parturients within the age range of 18-22 years were positive for placenta parasitaemia.

**Table 2.** Demographic characteristics of the parturients associated with risk of placental malaria.

Risk factor	Number (%) examined	+ve malaria for placental *N* (%)	OR (95% CL)*	*P*-value
**Age (years)**				**0.046**
18 to 22	22 (10.4)	7 (31.8)	4.4 (1.1-17.4)	
23 to 27	53 (25.1)	11 (20.8)	2.5 (0.7-8.5)	
28 to 32	94 (44.6)	18 (19.1)	2.3 (0.7-7.1)	
33 and above	42 (19.9)	4 (9.5)	Referent	
**Gravidity**				**0.028**
Primigravidae	67 (31.8)	19 (28.4)	2.1 (0.9-5.1)	
Secundigravidae	57 (27.0)	9 (15.8)	Referent	
Multigravidae	87 (41.2)	12 (13.8)	0.9 (0.3-2.2)	
**Education**				**0.724**
Primary	39 (18.5)	9 (23.1)	1.3 (0.5-3.2)	
Secondary	77 (36.5)	13 (16.9)	0.69 (0.4-1.9)	
Tertiary	95 (45.0)	18 (18.9)	Referent	
**Type of Apartment**				**0.029**
Single room	85 (40.3)	28 (25.7)	1.6 (0.4-6.0)	
Flat	17 (8.1)	9 (10.6)	0.6 (0.1-2.3)	

* OR = Odds ratio; CL = Confidence limits

**Figure 1.**
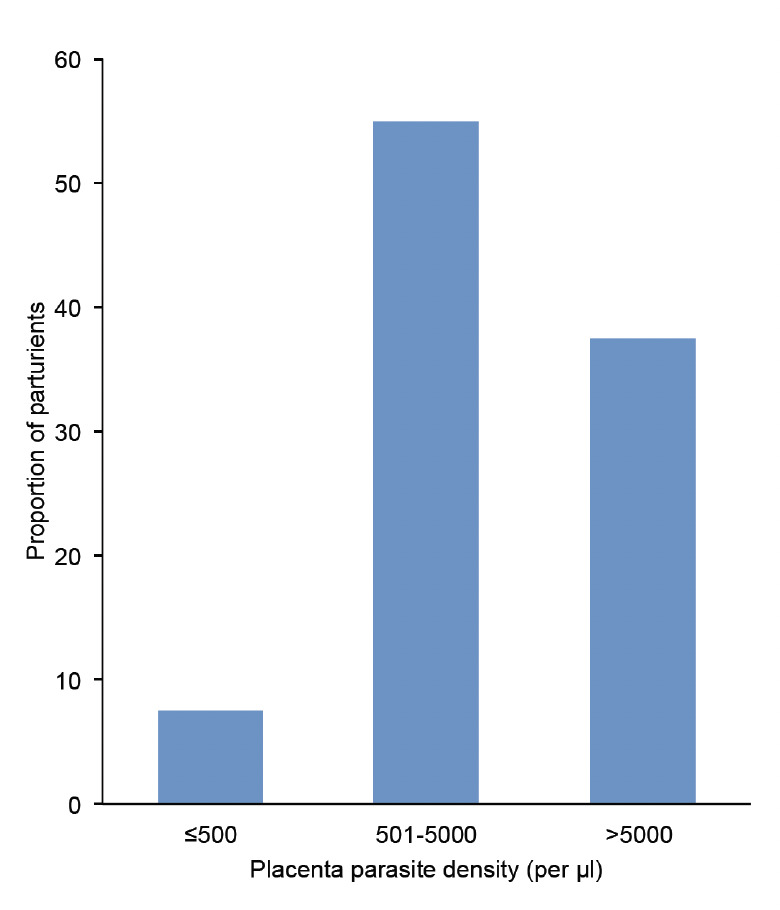
Parasite density in the placenta of 40 parturients with placental malaria.

The highest prevalence of placental malaria infection (28.4%) was detected among the primigravidae. Placental malaria parasite densities ranged from 26 parasites/μl of blood (i.e. low) to 15375 parasites/μl of blood (high), with the highest proportion showing moderate infection (501 – 5000) parasite/μl of blood (Figure 1).

### 3.3 Risk factors for placental malaria

Using univariate analysis for demographic information, we identified being within the age range 18-22 years [OR = 4.4, 95% CL = 1.1-17.4, *P* = 0.046], being primigravid [OR = 2.1, 95% CL = 0.9-5.1, *P* = 0.028] and living in a single-room apartment [OR = 1.6, 95% CL = 0.4-6.0, *P* = 0.029] as significant risk factors for placental malaria. Women with primary education recorded the highest prevalence (23.1%) of placental malaria compared with women with secondary (16.9%) and tertiary (18.9%) education (Table 2) although this was not significant [OR = 1.3, 95% CL = 0.5-3.2, *P* = 0.724].

Non-usage of intermittent preventive treatment (IPT) was found to be a significant risk factor for placental malaria [OR = 2.6, 95% CL = 1.2-5.4, *P* = 0.018], since a higher proportion of the parturients (34.0%) who did not use IPT during pregnancy tested positive for placental malaria infections (Table 3). We also identified non-usage of LLINs as a significant risk factor for placental malaria [OR = 2.7, 95% CL = 1.3-5.5, *P* = 0.005]. Sharing a room with more than one person during pregnancy was also identified as a significant risk factor (*P* = 0.043) [OR = 2.2, 95% CL = 1.0-4.6], since a higher proportion of the parturients (29.2%) who claimed to have a shared room with more than one person tested positive for placental malaria (Table 3).

**Table 3.** Risk factors for placental malaria among parturients.

Risk factors	Number (%) examined	+ve malaria for placental *N* (%)	OR (95% CL)*	*P*-value
**IPT usage**				**0.018**
Yes	160 (77.3)	24 (15.0)	Referent	
No	47 (22.3)	16 (34.0)	2.6 (1.2-5.4)	
**LLIN usage**				**0.005**
Yes	126 (59.7)	16 (12.7)	Referent	
No	85 (40.3)	24 (28.2)	2.7 (1.3-5.5)	
**Sharing of room**				**0.043**
Yes	48 (22.7)	14 (29.2)	2.2 (1.0-4.6)	
No	163(77.3)	26 (16.0)	Referent	
**With maternal malaria**				**0.002**
Yes	86 (40.8)	25 (29.1)	3.0 (1.5-6.1)	
No	125 (59.2)	15 (12.0)	Referent	
**Malaria episodes during pregnancy**				**0.080**
Yes	70 (33.2)	18 (25.7)	1.9 (0.9-3.8)	
No	141 (66.8)	22 (15.6)	Referent	
**Screened housing**				**0.139**
Yes	202 (95.7)	40 (19.8)	0.234 (0.00-1.89)	
No	9 (4.3)	0 (0.0)	Referent	

* OR = Odds ratio; CL = Confidence limits

The presence of malaria parasite infections in maternal peripheral blood at delivery as a significant risk factor (*P* = 0.002) for placental malaria [OR = 3.0, 95% CL = 1.5-6.1]. There was no significant difference among the parturients that suffered from one or more episodes of malaria infections during pregnancy and those that did not (*P*>0.05). However, the univariate analysis revealed that the risk of placental malaria infections among this category of parturients was twice as high as in those who did not suffer any malaria attack during pregnancy [OR = 1.9, 95% CL = 0.9-3.8, *P* = 0.080]. Finally, the non-usage of window- and door screening was not a significant risk factor (*P* = 0.139). All parturients who tested positive for placental malaria claimed to be residing in screened housing even before pregnancy.

### 3.4 Health-seeking behaviour and occurrence of placental malaria

The majority of parturients exercised good health-seeking behaviours by visiting the hospital when they were sick during pregnancy, while the rest used either herbs or drugs prescribed by vendors (poor health-seeking behaviour). All parturients that treated malaria episodes during pregnancy with anti-malaria injections tested positive for both maternal and placental malaria infections (Table 4). Furthermore, the highest prevalence of placental malaria parasite infections was recorded among parturients who treated malaria infections (during pregnancy) with injections, with a prevalence of 3 (100.0%). This was followed by respondents that claimed they did not remember the drugs they used, as they recorded a prevalence of 75% for both maternal and placental malaria infections (Table 4). It is noteworthy, as stated by the respondents, that the antimalaria injections were prescribed by attendants in ‘chemist’s shops’. Those who did not remember the drugs they used claimed that the drugs were prescribed to them from ‘chemist’s shops’ (Table 4).

**Table 4.** Health seeking behaviour and occurrence of maternal and placental malaria infections in 70 parturients.

Behaviour types	No. examined *N* (%)	Number (%) with maternal peripheral malaria	Number (%) with placental malaria
**Malaria treatment during pregnancy**			
Anti-malaria drugs	47 (67.1)	15 (31.9)	9 (19.1)
Herbs	8 (11.4)	2 (25.0)	2 (25.0)
Placed on hospital admission	8 (11.4)	5 (62.5)	1 (12.5)
Anti-malaria injection*	3 (4.3)	3 (100.0)	3 (100.0)
Could not remember*	4 (5.8)	3 (75.0)	3 (75.0)
	***P*-value**	**0.036**	**0.004**
**Treatment prescribed by whom?**			
Doctor	55 (78.6)	20 (36.4)	12 (21.8)
Chemist	7 (10.0)	6 (85.7)	5 (71.4)
Self medication	7 (10.0)	2 (28.6)	1 (14.3)
Native doctor	1 (1.4)	0 (0.0)	0 (0.0)
	***P*-value**	**0.059**	**0.03**

* Treatment administered by chemist attendants or drug vendors, rather than doctors

### 3.5 Challenges associated with IPT usage

Almost half of the parturients (49.0%) claimed not to use IPT, due to fear of side effects, whereas some complained about the unavailability of the drug during their first and second antenatal visits. Furthermore, some of the respondents claimed not to attach any importance to IPT usage.

## 4 Discussion

The epidemiology of placental malaria in Abeokuta Ogun state, Nigeria is similar to that in other parts of Nigeria [6,15]. In the current study, women within the age group of 18-22 years as well as primigravidae were more likely to have placental malaria.

While the risk of placenta infections is twice as high in primigravidae compared to other gravidities, placental malaria seems to increase fourfold among parturients between the age range of 18-22 years compared with other age groups. This could be attributed to lower levels of acquired immunity among the young women as well as lack of pregnancy-associated and naturally acquired immunity against the placenta-binding parasites in primigravids. Studies have shown that immunity against the placenta-binding parasites develops with subsequent pregnancies as a result of previous exposure to the placenta binding strain [18,19].

Living in a single-room apartment as well as the sharing of a room with more than one person during pregnancy were found to be significant risk factors for placental malaria. This could be related to overcrowding and the frequency of times household doors are opened, which in turn increases the number of mosquitoes that could gain access to the house [20]. Studies have also shown that malaria infection can increase with overcrowding, as mosquitoes are attracted to higher levels of carbon dioxide and other body odours present in crowded houses [21].

Clinical malaria during pregnancy and maternal malaria parasite infection at parturition increased the risk for placental malaria, with a twofold (non-significant) increase in risk for the former and threefold increase in risk for the latter. In a study carried out in eastern Nigeria, Ezebialu *et al.* [15] reported similar findings. Pregnant women who had taken precautions and preventive measures during pregnancy had reduced placenta parasitisation, thereby minimizing the effect of malaria parasite infections during pregnancy.

The risk of placental malaria parasite infection in the current study was threefold higher among the parturients that did not use IPT and LLIN, respectively, during pregnancy. Several studies had reported similar findings [18,22,23]. However, it is saddening to note that despite the effectiveness of IPT and its administration as directly observed therapy (DOT), most of the parturients that failed to use IPT during pregnancy claimed that they were given IPT, but only pretended to swallow it during their antenatal visits. This situation justifies the need to stress the importance of IPT and LLIN as preventive tools during pregnancy in order to improve compliance.

This study also suggests that the use of window- and door screening might not be very effective in the prevention of malaria infection, due to a number of reasons as stated by Idowu *et al.* [24], who discovered that the majority of the screens in their study were torn by children or during the process of passing a wire for external antennas, thereby facilitating entry of mosquitoes.

The health-seeking behaviour of women with regards to malaria infection during pregnancy also has an association with susceptibility to placental malaria, as pregnant women that seek treatment with unqualified and untrained ‘chemist’s shops’ recorded the highest prevalence of placental malaria infection. Studies have shown that most of the anti-malarial drugs acquired from chemist shops are of sub-standard quality [25] that have little or no effect against malaria. This practice can significantly increase the risk of placental malaria.

## 5 Conclusions

In Abeokuta, approximately one in every five parturients had placental malaria at delivery, with 55% having parasite densities between 501 and 5000 parasites/μl of blood. Women within the age range of 18-22 years and primigravidae were at the highest risk of placental malaria. Other factors that contribute to the risk of placenta infections included non-usage of LLIN and IPT, poor housing conditions and poor health-seeking behaviour.
